# Rare Presentation of Postpericardiotomy Syndrome After Left Atrial Myxoma Removal

**DOI:** 10.1177/23247096231217858

**Published:** 2023-12-17

**Authors:** Jessy Feng, Nina Shyama Appareddy, Yaman Gibran, Aryana Garza, Jacqueline Luevano, Gustavo Garcia

**Affiliations:** 1 University of Texas Rio Grande Valley School of Medicine, Edinburg, USA; 2 Valley Baptist Medical Center, Harlingen, TX, USA

**Keywords:** myxoma, pericardial effusion, anticoagulation, postpericardiotomy syndrome

## Abstract

Postpericardiotomy syndrome (PPS) is a known complication of cardiac valve surgery, but it has not been commonly reported as a postoperative complication of cardiac myxoma removal. A 78-year-old female with hypertension and atrial fibrillation presenting with angina was found to have a large left atrial myxoma (7.5 cm × 4.4 cm). The myxoma was resected; however, 1-week postoperation hemoglobin and blood pressure decreased with elevated erythrocyte sedimentation rate (ESR) and C-reactive protein (CRP). Limited transthoracic echocardiogram (TTE) showed moderate pericardial effusion, confirming the diagnosis of PPS. This case highlights the importance of monitoring patients postremoval of myxoma for symptoms of PPS.

## Introduction

Postcardiac injury syndrome (PCIS), previously known as Dressler syndrome, comprises autoimmune-mediated pericarditis conditions with risk factors that include myocardial infarction, chest trauma, or cardiac surgery. Postpericardiotomy syndrome (PPS) is a subgroup of PCIS characterized by pericarditis symptoms that may emerge 1 to 6 weeks after cardiac surgery. Here, we report a rare case of PPS postremoval of a left atrial myxoma.

## Case Report

**Figure 1.**
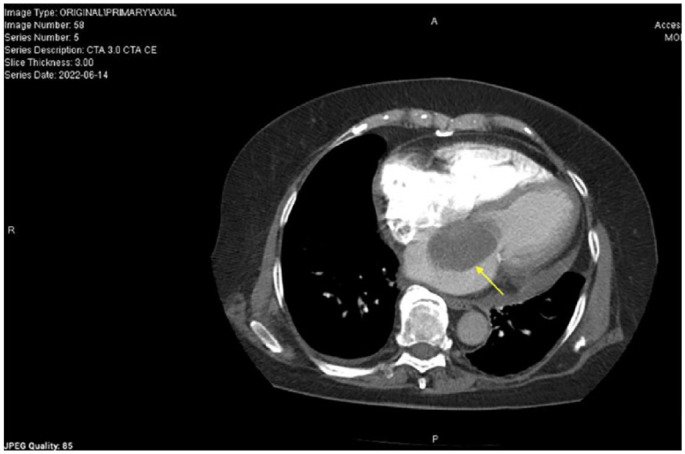
Computed tomography angiography chest of left atrial myxoma (yellow arrow).

**Figure 2.**
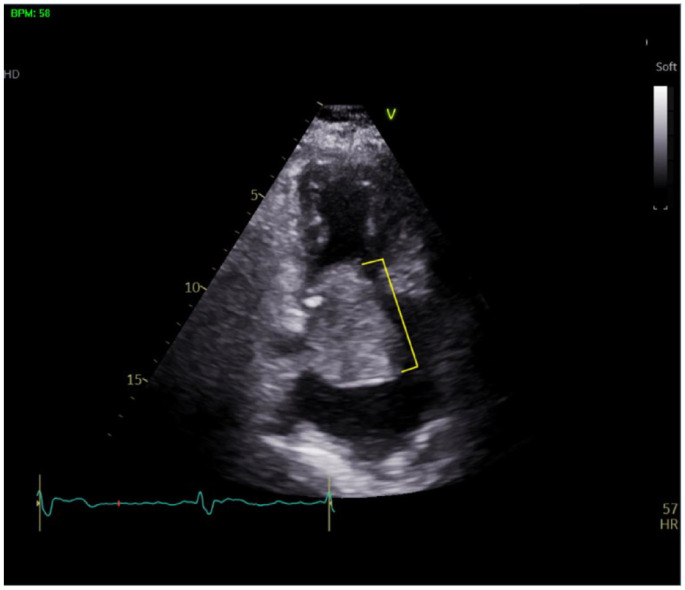
Transthoracic echocardiogram of left atrial myxoma (yellow bracket).

A 78-year-old Hispanic female with a history of hypertension and atrial fibrillation (AF) CHA2DS2-VASc 4/HAS-BLED 3/ORBIT 4 on dabigatran presented to the emergency department (ED) with anginal symptoms. Troponins were negative, electrocardiogram (EKG) showed AF with controlled ventricular rate and no ischemic changes. D-dimer was elevated (Table 1). Subsequent computed tomography (CT) PE did not show evidence of pulmonary embolism, but revealed a large left atrial mass consistent with myxoma (Figure 1). Initial transthoracic echocardiogram (TTE) showed severe left atrial dilatation, atrial myxoma measuring 7.5 cm × 4.4 cm, and small pericardial effusion (Figure 2, Table 2). The myxoma caused secondary mitral valve dysfunction, acute-on-chronic diastolic heart failure (left ventricular ejection fraction (LVEF) 55-60%) and intermittently compressed her coronary arteries causing anginal symptoms. Cardiology conducted a left heart catheterization which showed large left atrial branches feeding the myxoma. Computed tomography surgery resected the left atrial myxoma via pericardiotomy and ligated the left atrial appendage, reducing the risk of stroke.

**Table 1.** Admission Lab Results.

Result	Value	Reference range
Complete blood count		
White blood cells (WBC)	8.9	3.2-10 cells/mm^3^
Hemoglobin (HGB)	10.7	11-15 g/dL
Mean corpuscular volume (MCV)	76.3	79-97 fL
Red distribution width (RDW)	17.9%	11-16%
Platelets	292	125-390 × 10^9^/L
Eosinophil relative	6.2%	0-6%
Immature granulocytes	0.7%	0-0.5%
Complete metabolic panel		
Glucose	110	70-105 mg/dL
Sodium	133	136-145 meq/L
Potassium	4.8	3.5-5.1 meq/L
Chloride	104	98-107 meq/L
CO_2_	23	21-31 meq/L
Calcium	7.7	8.6-10.3 mg/dL
Bun	25	7-25 mg/dL
Creatinine	0.9	0.6-1.2 mg/dL
Bun/creatinine	28	10-20
Albumin	3	3.5-5.7 g/dL
Alkaline phosphatase	62	34-104 U/L
Alanine transaminase	16	7-52 U/L
Aspartate transaminase	17	13-29 U/L
Lipase	95	11-82 U/L
Estimated glomerular filtration rate (eGFR)	65	≥90 mL/min
Bilirubin total	0.3	0.3-1 mg/dL
Total protein	5.9	6.4-8.9 g/dL
Urinalysis		
Specific gravity	1.039	1.005-1.030
Protein	10	Negative
Leukocyte esterase	75	Negative
Glucose	50	Negative
WBC	23	0-1 WBCs/hpf
Other		
Brain natriuretic peptide (BNP)	308	<100 pg/mL
Troponin I	22	10-27 ng/L
COVID antigen	Negative	Negative
D-dimer	1.26	≤50
Vitamin B12	>100 000	
International normalied ratio (INR)	1.29	0.9-1.3
Prothrombin time (PT)	16.5	11.9-15.2 s
Partial thromboplastin time (PTT)	46.1	22-35.3 s

**Table 2.** Admission Echo.

Mitral valve (MV) area pressure half-time (PHT)	1.6 cm^2^
Mitral regurgitation (MR) peak velocity	344.3 cm/s
MR peak gradient	47.4 mm Hg
Mitral E point velocity	223.7 cm/s
Mitral A point velocity	96.9 cm/s
Mitral E to A ratio	2.3

Report: Large atrial myxoma seen measuring 7.5 cm × 4.4 cm with partial obstruction of left ventricle (LV) inflow, partial prolapse into the LV, and functional mitral valve stenosis. MV “area” by pressure 1/2 time method 1.6 cm^2^. Trace to mild mitral regurgitation. Trivial pericardial effusion seen anteriorly.

Cardiac electrophysiology (EP) then performed a transesophageal echocardiogram–guided cardioversion with prompt reversion to AF, which may have been due to inflammation from the recent pericardiotomy. Amiodarone 200 mg QD, metoprolol 12.5 mg BID, and Eliquis 5 mg QD were continued with a 1-month follow-up to reconsider cardioversion after postoperative inflammation subsided.

**Figure 3.**
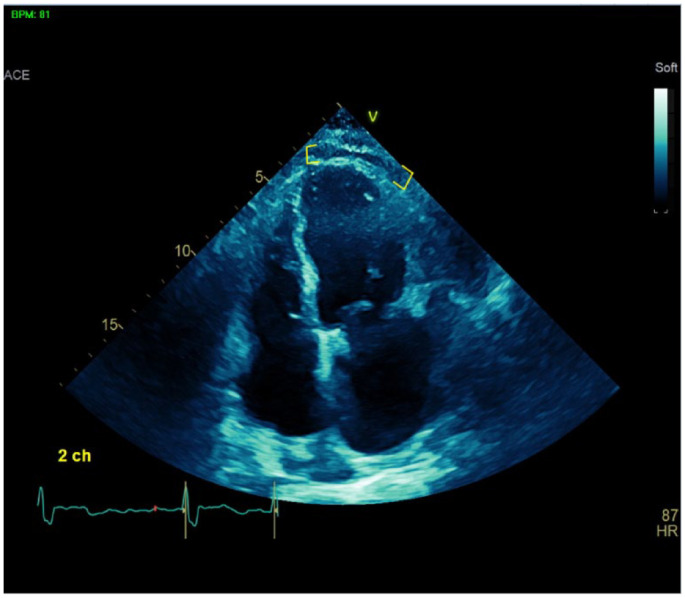
Transthoracic echocardiogram of pericardial effusion (yellow brackets) postremoval of left atrial myxoma.

Perioperatively, the patient received several blood transfusions, otherwise remaining stable. One week postoperatively, her hemoglobin and blood pressure decreased without overt signs of bleeding. Computed tomography chest showed 2 mediastinal hematomas and suggested a moderate pericardial effusion. With incidental pericardial effusion on admission, a limited TTE was repeated and found a moderate pericardial effusion consistent with pericarditis (Figure 3). Erythrocyte sedimentation rate was elevated at 40, and C-reactive protein (CRP) was elevated at 4.8 (Table 3). Given her new findings of moderate pericardial effusion with pericarditis, she was diagnosed with PPS and started on ibuprofen 600 mg TID and colchicine 0.6 mg BID. After extensive discussion, it was decided to temporarily hold anticoagulation for outpatient follow-up.

**Table 3.** Diagnosis of postpericardiotomy syndrome.

White blood cells (WBC)	8.1	3.2-10 cells/mm^3^
Hemoglobin (HGB)	8.3	11-15 g/dL
Platelets	355	125-390 × 10^9^/L
Erythrocyte sedimentation rate (ESR)	40	0-30 mm/h
Glucose	102	70-105 mg/dL
Sodium	134	136-145 meq/L
Potassium	4	3.5-5.1 meq/L
Chloride	98	98-107 meq/L
CO_2_	32	21-31 meq/L
Calcium	7.9	8.6-10.3 mg/dL
Bun	21	7-25 mg/dL
Creatinine	0.8	0.6-1.2 mg/dL
Bun/creatinine	26	10-20
Estimated glomerular filtration rate (eGFR)	70	≥90 mL/min
C-reactive protein (CRP) quantitative	4.8	<1 mg/dL
Magnesium	2.1	1.9-2.7 mg/L
Phosphate	3.1	2.5-5 mg/dL

Abbreviation: CRP: C-reactive protein.

## Discussion

Myxomas are the most common primary cardiac tumor in adults, with over 75% of cases originating in the left atrium.^1^ Postpericardiotomy syndrome is characterized by 2 out of the 5 criteria: fever, anterior chest pain worsened with inspiration and supine position, pericardial friction rub, pericardial effusion, and pleural effusion with elevated CRP.^2,3^ It is a common complication of cardiac surgery occurring in about 10% of patients, most commonly after aorta, aortic valve, and mitral valve surgery.^4,5^ However, PPS has not been commonly reported as a complication postremoval of cardiac myxomas.^6-8^ Studies conducted to identify risk factors of developing PPS among patients undergoing coronary artery bypass graft (CABG) and valve replacements found increased risk with younger age, lower platelet counts, reduced body weight, blood transfusions, and renal dysfunction.^2,9^ Our patient received several blood transfusions postoperatively, which may have increased her risk for developing PPS.

Management of PPS is similar to idiopathic pericarditis with nonsteroidal anti-inflammatory drugs (NSAIDs) and colchicine. The COPPS-2 trial investigated the perioperative use of colchicine placebo among cardiac surgery patients and saw a reduction in the incidence of PPS compared with placebo.^10^ Although no patients in the trial underwent pericardiotomy specifically for cardiac myxomas, it may be worth considering administering prophylactic colchicine to myxoma surgery patients to prevent PPS. It is worth addressing that the trial demonstrated a 2-fold increase of gastrointestinal adverse effects, 11.7% in the placebo group versus 20.0% in the colchicine group, and similar discontinuation rates among the two groups, which suggests that colchicine pretreatment may not be applicable to all patients. Future studies should be conducted to explore the risk factors linked to PPS in patients who have undergone cardiac myxoma removal to facilitate more effective identification of these patients, which gives the treatment team a chance to decide if the patient is a good candidate for colchicine prophylaxis.

The American College of Cardiology published an expert analysis in 2015 which reviewed studies looking at prophylactic treatments for PPS.^11^ There was a study of 1019 adult patients who underwent cardiac surgery which showed taking 600 mg of ibuprofen QID or 24 mg indomethacin QID for 10 days was effective in reducing the incidence of PPS.^12^ However, there was also a retrospective study of 177 pediatric patients who underwent cardiac surgery which found that aspirin did not reduce the incidence of PPS.^13^ Thus, there is no consensus on standard of care prophylaxis at the moment for PPS. Given the potential positive research findings for ibuprofen or colchicine for PPS prophylaxis, it would be beneficial to have a discussion about prophylaxis in patients similar to our case who are undergoing surgery for myxoma removal.

Some challenges with diagnosing our patient were the confounding chest pain postsurgery as it was difficult to determine if the pain was attributed to PPS or the postsurgery healing process. However, additional labs such as ESR and CRP and imaging can aid the diagnosis of PPS. Our case highlights the potential for PPS to develop in patients who have undergone cardiac myxoma removal. Prophylactic treatment should be discussed for every patient undergoing cardiac myxoma removal surgery. Following surgery, patients should be closely monitored for the emergence of symptoms consistent with PPS.
